# Technology and Continuous Glucose Monitoring Access, Literacy, and Use Among Patients at the Diabetes Center of an Inner-City Safety-Net Hospital: Mixed Methods Study

**DOI:** 10.2196/54223

**Published:** 2024-10-15

**Authors:** Gaëlle Sabben, Courtney Telfort, Marissa Morales, Wenjia Stella Zhang, Juan C Espinoza, Francisco J Pasquel, Kate Winskell

**Affiliations:** 1 Hubert Department of Global Health Rollins School of Public Health Emory University Atlanta, GA United States; 2 Center for the Study of Human Health College of Arts and Sciences Emory University Atlanta, GA United States; 3 Division of Hospital Based Medicine Department of Pediatrics Ann & Robert H Lurie Children’s Hospital of Chicago Chicago, IL United States; 4 Division of Endocrinology Department of Medicine Emory University School of Medicine Atlanta, GA United States

**Keywords:** diabetes mellitus, type 1, type 2, digital health, continuous glucose monitoring, mobile phone

## Abstract

**Background:**

Despite the existence of an increasing array of digital technologies and tools for diabetes management, there are disparities in access to and uptake and use of continuous glucose monitoring (CGM) devices, particularly for those most at risk of poor diabetes outcomes.

**Objective:**

This study aims to assess communication technology and CGM access, literacy, and use among patients receiving treatment for diabetes at an inner-city safety-net hospital.

**Methods:**

A survey on digital technology ownership and use was self-administered by 75 adults with type 1 and type 2 diabetes at the diabetes clinic of Grady Memorial Hospital in Atlanta, Georgia. In-depth interviews were conducted with 16% (12/75) of these patient participants and 6 health care providers (HCPs) to obtain additional insights into the use of communication technology and CGM to support diabetes self-management.

**Results:**

Most participants were African American (66/75, 88%), over half (39/75, 52%) were unemployed or working part time, and 29% (22/75) had no health insurance coverage, while 61% (46/75) had federal coverage. Smartphone ownership and use were near universal; texting and email use were common (63/75, 84% in both cases). Ownership and use of tablets and computers and use and daily use of various forms of media were more prevalent among younger participants and those with type 1 diabetes, who also rated them as easier to use. Technology use specifically for diabetes and health management was low. Participants were supportive of a potential smartphone app for diabetes management, with a high interest in such an app helping them track blood sugar levels and communicate with their care teams. Younger participants showed higher levels of interest, perceived value, and self-efficacy for using an app with these capabilities. History of CGM use was reported by 56% (42/75) of the participants, although half (20/42, 48%) had discontinued use, above all due to the cost of the device and issues with its adhesive. Nonuse was primarily due to not being offered CGM by their HCP. Reasons given for continued use included convenience, improved blood glucose control, and better tracking of blood glucose. The in-depth interviews (n=18) revealed high levels of satisfaction with CGM by users and supported the survey findings regarding reasons for continued use. They also highlighted the value of CGM data to enhance communication between patients and HCPs.

**Conclusions:**

Smartphone ownership was near universal among patients receiving care at an inner-city hospital. Alongside the need to address barriers to CGM access and continued use, there is an opportunity to leverage increased access to communication technology in combination with CGM to improve diabetes outcomes among underresourced populations.

## Introduction

### Background

There has been a marked acceleration in the use of digital communication technology to deliver care since the COVID-19 pandemic [1]. This has further fueled concern that people with diabetes lacking access to and literacy in such technologies may be left behind in an expanding digital diabetes ecosystem [2,3].

Continuous glucose monitoring (CGM) is recognized as an important diabetes management tool leading to clinically meaningful reductions in glycated hemoglobin even among patients with type 2 diabetes (T2D) on less intensive insulin regimens [4]. However, the known benefits of CGM are not evenly distributed, with underresourced populations, particularly African American individuals [5], having lower rates of CGM use and higher rates of discontinuation [6-9].

Several CGM systems have their own proprietary smartphone apps that can be used as an alternative to a stand-alone CGM reader to display CGM data and, like readers, alert patients via alarms to highs and lows [10]. Meta-analyses also suggest that digital apps can facilitate and optimize self-care among people with diabetes [11,12], and some commercial diabetes apps have the capacity to link to popular CGM systems [13,14].

A recent Pew report indicates a marked increase in US cellphone ownership (to approximately 97%) regardless of racial, ethnic, or socioeconomic background. While smartphone ownership has also increased (to approximately 85%), this is lower among older, less educated, and lower-income Americans [15]. A recent survey among vulnerable, primarily Latino, community residents in East Harlem, New York, suggests that the digital divide in access to communication technology is narrowing, and study participants expressed interest in health-promoting apps [16]. However, diabetes health apps are often not accessible to populations vulnerable to the worst diabetes outcomes, namely, minoritized populations with the lowest socioeconomic status and the least education [17,18]. Barriers to access to these apps may include platform (access to recent models of smartphones and data plans); affordability (paywalls or other restrictions); lack of integration with health care; and technology, data, and health literacy (excessive complexity or user burden and limited appeal) [19-21].

### Objectives

To explore the feasibility of an app for underresourced patients with type 1 diabetes (T1D) and T2D, we assessed communication technology access, use, and literacy along with CGM use and discontinuation patterns by means of a self-administered survey among patients receiving care at the diabetes center of an inner-city safety-net hospital. We additionally conducted in-depth interviews (IDIs) with a subset of those patient participants and their health care providers (HCPs) to gain insights into their experiences with CGM and app use for diabetes management.

## Methods

### Overview

Patient participants and HCPs were recruited from the Grady Diabetes Center (GDC) at Grady Memorial Hospital, Atlanta, Georgia.

For the survey portion of the study, efforts were made to ensure recruitment of individuals with and without previous exposure to CGM, with T1D and T2D, as well as male and female participants. Individuals with impaired decision-making capacity or inability to provide consent, including due to an English-language barrier, and minors were excluded from participation. Pregnant individuals were eligible as long as they had a T1D or T2D diagnosis. Data collection took place between June 2021 and December 2021, during which time we enrolled 75 consecutive adult patients currently receiving care at the GDC and its monthly or bimonthly Technology Clinic. Recruitment was first initiated through phone-based contact of individuals with a CGM account linked to the Grady LibreView practice. LibreView is a cloud-based system that allows HCPs to view reports summarizing patients’ glucose readings from the FreeStyle Libre CGM system. In response to the limited success of this method (only 4 participants were identified through this strategy), it was replaced with waiting room–based direct contact. Trained study staff members screened participants for eligibility and secured consent either in person at the clinic or by phone. Participants self-administered the survey via tablet-based REDCap (Research Electronic Data Capture; Vanderbilt University) [22] unless they requested study staff assistance in navigating the survey. Questions included ownership and use of communication technology devices (smartphones, tablets, computer, and wearables) and use of media (texting, mobile apps, email, social media, podcasts, web videos, and websites). These items were drawn and adapted from the Technology Access and Competency Scale, an instrument developed by author JE and colleagues that has not undergone psychometric validation but has been used in other technology-related studies [23,24]. Items related to interest in using and intention to use technology for diabetes self-management [25] were also included. Additional demographic (including insurance status) and biometric data were extracted from electronic medical records and used to confirm self-reported diabetes diagnosis (ie, T1D vs T2D). Descriptive statistics (counts and proportions or means and SDs, as appropriate) were calculated for demographics and technology use and access items using the SAS software (version 9.4; SAS Institute). These were also stratified by diabetes type and age group to elucidate potential within-sample differences.

IDIs were conducted with 12 patient participants and 6 HCPs. Survey participants who indicated willingness to take part in an IDI and met additional eligibility criteria (current or previous CGM use) were contacted for participation in this portion of the study and enrolled consecutively. IDIs focused on patients’ experiences with CGM and their use of technology tools for diabetes self-management. HCP IDI participants were selected purposively from among HCPs working at the GDC to cover a range of specializations. These IDIs focused on HCPs’ perspectives on their patients’ use of technology, particularly CGM, for diabetes self-management and its impact thereon. All IDIs were conducted via Zoom (Zoom Video Communications) and lasted between 30 and 75 minutes. Transcripts of audio recordings were reviewed for accuracy and deidentified before being uploaded into MAXQDA 2022 (VERBI GmbH) for coding and analysis. A codebook of deductive themes focusing on domains addressed in the IDI guides and inductive themes emerging from the transcripts was systematically applied to the transcripts. Thematic analysis of coded segments focused on the challenges of diabetes self-management and opportunities offered by digital technology, along with benefits and difficulties associated with CGM system use as perceived by both patient participants and HCPs. Data were stratified by interviewee group.

### Ethical Considerations

This study was approved by the Emory University Institutional Review Board and Grady Memorial Hospital’s Research Oversight Committee (IRB#00002376). All participants in the survey and IDI study components were compensated for their time at a rate of US $10 for survey completion and US $25 for IDI participation.

## Results

### Participant Demographics

Among survey respondents, most were African American (66/75, 88%), and two-thirds were female (50/75, 67%; Table 1). Half (37/75, 49%) had a high school or General Educational Development diploma or a lower educational level; 17% (13/75) were working full time, with 28% (21/75) being retired and 40% (30/75) being unemployed or not working. A total of 55% (41/75) had T2D, and 83% (62/75) reported current insulin use. Most (46/75, 61%) had federal insurance coverage, including Medicare and Medicaid; 29% (22/75) had no insurance coverage.

**Table 1:** Survey respondent demographics (n=75).

			Total, n (%)		Type 1 diabetes (n=34), n (%)		Type 2 diabetes (n=41), n (%)		Aged <45 years (n=27), n (%)		Aged 45-59 years (n=25), n (%)		Aged >59 years (n=23), n (%)
Female			50 (67)		21 (62)		29 (71)		17 (63)		18 (72)		15 (65)
African American			66 (88)		33 (97)		33 (80)		26 (96)		21 (84)		19 (83)
**Educational level**													
	High school diploma or GED^a^ or lower	37 (49)		13 (38)		24 (59)		12 (44)		14 (56)		11 (48)	
	Some college	21 (28)		13 (38)		8 (20)		10 (37)		6 (24)		5 (22)	
	College degree	11 (15)		6 (18)		5 (12)		4 (15)		2 (8)		5 (22)	
	Advanced degree	6 (8)		2 (6)		4 (10)		1 (4)		3 (12)		2 (9)	
**Employment status**													
	Retired	21 (28)		4 (12)		17 (41)		1 (4)		3 (12)		17 (74)	
	Working full time	13 (17)		10 (29)		3 (7)		9 (33)		3 (12)		1 (4)	
	Working part time	9 (12)		6 (18)		3 (7)		5 (19)		4 (16)		0 (0)	
	Unemployed or not working	30 (40)		12 (35)		18 (44)		10 (37)		15 (60)		5 (22)	
	Student	2 (3)		2 (6)		0 (0)		2 (7)		0 (0)		0 (0)	
**Diabetes diagnosis**													
	Type 1	34 (45)		34 (100)		—^b^		25 (93)		7 (28)		2 (9)	
	Type 2	41 (55)		—		41 (100)		2 (7)		18 (72)		21 (91)	
Insulin use			62 (83)		34 (100)		28 (68)		27 (100)		21 (84)		14 (61)
**Insurance coverage type**													
	Federal	46 (61)		19 (56)		27 (66)		15 (56)		13 (52)		18 (78)	
	Private	7 (9)		4 (12)		3 (7)		2 (7)		4 (16)		1 (4)	
	None	22 (29)		11 (32)		11 (27)		10 (37)		8 (32)		4 (17)	

^a^GED: General Educational Development.

^b^Not applicable.

### Technology Use, Access, and Literacy

#### Survey Findings

Smartphone ownership and use were near universal (71/75, 95%), and 88% (66/75) reported using a smartphone daily (Table 2). Most participants (42/75, 56%) had Android devices, and 84% (54/64) of those who provided a model name that could be dated owned models that had become available in the last 5 years. A total of 89% (67/75) of the participants accessed the internet on their phones, 73% (55/75) had unlimited data plans, and 93% (70/75) had Wi-Fi access at home. Ownership and daily use of devices was generally highest among younger participants and those with T1D, who also rated all devices as easier to use than their older counterparts and those with T2D. Smartphones were deemed the easiest to use (68/75, 91% found them “very easy” or “somewhat easy” to use), and wearables were deemed the least easy to use (21/75, 28%).

Of the media types presented in the survey (texting, mobile apps, email, social media, podcasts, web videos, and websites), texting was the medium most commonly used (63/75, 84%), and 73% (46/63) of texters indicated that they used it daily (Table 3). Email use was as common as texting, although it was used less frequently. Social media was used by 65% (49/75) of participants, 63% (31/49) of whom reported daily use. Over 50% of participants reported use of web videos (43/75, 57%), mobile apps (53/75, 71%), and websites (55/75, 73%). Podcasts were used by a minority of participants, and daily use was very low. In general, use and daily use of media was highest among younger participants; however, a larger proportion of participants aged >59 years used email daily than their counterparts aged 45 to 59 years; daily video use was similar across age groups. Daily use of media was more common among those with T1D (who were overall younger) for everything except videos, websites, and podcasts. With the exception of podcasts, all forms of media were rated as “somewhat easy” or “very easy” to use by at least 65% (49/75) of users. The 2 older age groups rated texting, email, and web videos as similarly easy to use, and websites notably scored better among the oldest age group than among the middle one.

Approximately 70% of the participants indicated that they used some type of device (53/75, 71%) and some form of media (51/75, 68%) to manage their health (Table 4). Despite a majority of participants (57/75, 76%) owning a computer or tablet, few reported using these for health or diabetes management. Participants with T1D used smartphones and wearables (not including CGM devices) for health and diabetes management more than those with T2D. The media tool most commonly used for health management was websites, used by 37% (28/75) of participants for both health and diabetes management, followed by email (25/75, 33% for health management and 22/75, 29% for diabetes management). Texting was used for diabetes or health management by 24% (18/75) and 25% (19/75) of the participants, respectively, and apps were used by 20% (15/75) of the participants for diabetes management and 27% (20/75) of the participants for general health management. Only 5% (4/75) of the participants reported leveraging social media for diabetes management, 9% (7/75) reported using it to manage their health, and none were in the oldest age group.

Most participants indicated that they would find an app to help them manage their diabetes interesting (58/75, 77%), helpful (58/75, 77%), and time saving (50/75, 67%), with a higher proportion of those with T1D than T2D responding positively (Table 5). Interest in and perception of this app as useful or interesting also decreased with age. Four-fifths (60/75, 80%) of the participants indicated that they would feel confident using a mobile app to help them with their diabetes, and a similar proportion (58/75, 77%) indicated that they would likely use such an app. Similarly to participants’ perception of the app, younger participants and those with T1D were more confident in their ability to use and their likelihood of using such an app than their older counterparts. While 93% (25/27) of participants aged <45 years indicated that they felt confident that they could use an app to help with their diabetes management, that proportion fell to 84% (21/25) in the middle age group and 61% (14/23) among those aged >59 years. The oldest age group was also the least likely to use an app to help them manage their diabetes, with just over half (12/23, 52%) indicating that they were likely to do so compared to 80% (20/25) of those aged 45 to 49 years and all but one of those in the youngest age group (26/27, 96%).

Features selected as being of interest included tracking blood sugar levels (64/75, 85%); communication with health professionals (60/75, 80%); diet (57/75, 76%) and physical activity (57/75, 76%) planning or tracking; SMS text message monitoring, notifications, and reminders (57/75, 76%); and communication with others who have diabetes (45/75, 60%). Some differences existed in participants’ interest in each proposed app feature by age and diabetes type. While all age groups expressed the most interest in an app that tracked blood sugar levels, physical activity planning or tracking was equally appealing to the youngest age group (25/27, 93%), whereas the middle age group indicated equal interest in blood sugar level tracking and communicating with health professionals (21/25, 84%). For the oldest age group, diet planning or tracking was tied for second place with “text message monitoring, notifications, and reminders” (17/23, 74%). For all age groups, the least attractive feature proposed was communicating with others who have diabetes. For those living with T1D, relative interest mirrored that of the youngest age group, whereas participants with T2D found diet planning or tracking as interesting as blood sugar tracking (34/41, 83%), followed by communicating with health professionals (31/41, 76%) and SMS text message monitoring, notifications, and reminders (30/41, 73%).

**Table 2:** Device ownership, daily use, and usability (n=75).

			Total, n (%)		Type 1 diabetes (n=34), n (%)		Type 2 diabetes (n=41), n (%)		Aged <45 years (n=27), n (%)		Aged 45-59 years (n=25), n (%)		Aged >59 years (n=23), n (%)
**Device ownership and daily use**													
	Smartphone ownership		71 (95)		33 (97)		38 (93)		25 (93)		25 (100)		21 (91)
	Daily use for smartphone owners^a^		66 (93)		32 (97)		34 (89)		24 (96)		22 (88)		20 (95)
	Tablet ownership		40 (53)		22 (65)		18 (44)		19 (70)		11 (44)		10 (43)
	Daily use for tablet owners^a^		12 (30)		6 (27)		6 (33)		7 (37)		2 (18)		3 (30)
	Computer ownership		53 (71)		28 (82)		25 (61)		22 (81)		17 (68)		14 (61)
	Daily use for computer owners^a^		8 (15)		5 (18)		3 (12)		5 (23)		1 (6)		2 (14)
	Wearable ownership		20 (27)		14 (41)		6 (15)		11 (41)		7 (28)		2 (9)
	Daily use for wearable owners^a^		10 (50)		9 (64)		1 (17)		6 (55)		3 (43)		1 (50)
**Device usability**													
	Smartphones very or somewhat easy to use		68 (91)		34 (100)		34 (83)		26 (96)		23 (92)		19 (83)
	Tablets very or somewhat easy to use		38 (51)		25 (74)		13 (32)		22 (81)		10 (40)		6 (26)
	Computers very or somewhat easy to use		48 (64)		28 (82)		20 (49)		23 (85)		14 (56)		11 (48)
	Wearables very or somewhat easy to use		21 (28)		15 (44)		6 (15)		12 (44)		7 (28)		2 (9)
	Internet access using smartphone		67 (89)		34 (100)		33 (80)		27 (100)		22 (88)		18 (78)
	Unlimited smartphone data plan		55 (73)		26 (76)		29 (71)		22 (81)		17 (68)		16 (70)
	**Location of Wi-Fi access**												
		Home	70 (93)	33 (97)		37 (90)		27 (100)		22 (88)		21 (91)	
		School	9 (12)	9 (26)		0 (0)		8 (30)		1 (4)		0 (0)	
		Work	20 (27)	15 (44)		5 (12)		14 (52)		4 (16)		2 (9)	
		Library	9 (12)	5 (15)		4 (10)		3 (11)		4 (16)		2 (9)	
		Friends’ or relatives’ homes	30 (40)	16 (47)		14 (34)		10 (37)		12 (48)		8 (35)	
		Coffee shops, restaurants, and other businesses with free Wi-Fi	25 (33)	15 (44)		10 (24)		10 (37)		10 (40)		5 (22)	

^a^The denominator for the percentages in these rows is the numerator from the row above.

**Table 3:** Media type use, daily use, and usability (n=75).

			Total, n (%)		Type 1 diabetes (n=34), n (%)		Type 2 diabetes (n=41), n (%)		Aged <45 years (n=27), n (%)		Aged 45-59 years (n=25), n (%)		Aged >59 years (n=23), n (%)
**Media use and daily use**													
	Texting users	63 (84)		31 (91)		32 (78)		24 (89)		22 (88)		17 (74)	
	Daily texting for users^a^	46 (73)		26 (84)		20 (62)		22 (92)		15 (68)		9 (53)	
	Mobile app users	53 (71)		28 (82)		25 (61)		23 (85)		15 (60)		15 (65)	
	Daily app use for users^a^	36 (68)		20 (71)		16 (64)		16 (70)		11 (73)		9 (60)	
	Email users	63 (84)		30 (88)		33 (80)		23 (85)		21 (84)		19 (83)	
	Daily email use for users^a^	37 (59)		19 (63)		18 (55)		15 (65)		11 (52)		11 (58)	
	Social media users	49 (65)		25 (74)		24 (59)		22 (81)		16 (64)		11 (48)	
	Daily social media use for users^a^	31 (63)		17 (68)		14 (58)		15 (68)		11 (69)		5 (45)	
	Podcast users	14 (19)		8 (24)		6 (15)		7 (26)		6 (24)		1 (4)	
	Daily podcast use for listeners^a^	2 (14)		1 (12)		1 (17)		1 (14)		1 (17)		0 (0)	
	Video users	43 (57)		19 (56)		24 (59)		16 (59)		15 (60)		12 (52)	
	Daily video use for users^a^	23 (53)		10 (53)		13 (54)		9 (56)		8 (53)		6 (50)	
	Website users	55 (73)		26 (76)		29 (71)		23 (85)		16 (64)		16 (70)	
	Daily website use for users^a^	26 (47)		12 (46)		14 (48)		11 (48)		4 (25)		11 (69)	
**Media usability**													
	Texting very easy or somewhat easy to use	64 (85)		32 (94)		32 (78)		25 (93)		20 (80)		19 (83)	
	Apps very easy or somewhat easy to use	51 (68)		30 (88)		21 (51)		24 (89)		15 (60)		12 (52)	
	Email very easy or somewhat easy to use	66 (88)		33 (97)		33 (80)		27 (100)		20 (80)		19 (83)	
	Social media very easy or somewhat easy to use	50 (67)		27 (79)		23 (56)		23 (85)		16 (64)		11 (48)	
	Podcasts very easy or somewhat easy to use	20 (27)		13 (38)		6 (15)		11 (41)		7 (28)		1 (4)	
	Web videos very easy or somewhat easy to use	49 (65)		24 (71)		25 (61)		19 (70)		16 (64)		14 (61)	
	Websites very easy or somewhat easy to use	57 (76)		28 (82)		29 (71)		24 (89)		16 (64)		17 (74)	

^a^The denominator for the percentages in these rows is the numerator from the row above.

**Table 4:** Device and media type use for health and diabetes management (n=75).

			Total, n (%)		Type 1 diabetes (n=34), n (%)		Type 2 diabetes (n=41), n (%)		Aged <45 years (n=27), n (%)		Aged 45-59 years (n=25), n (%)		Aged >59 years (n=23), n (%)
**Device use for health and diabetes management**													
	Smartphone use for health	51 (68)		26 (76)		25 (61)		19 (70)		16 (64)		16 (70)	
	Smartphone use for diabetes	51 (68)		28 (82)		23 (56)		21 (78)		16 (64)		14 (61)	
	Tablet use for health	7 (9)		3 (9)		4 (10)		3 (11)		1 (4)		3 (13)	
	Tablet use for diabetes	7 (9)		3 (9)		4 (10)		3 (11)		1 (4)		3 (13)	
	Computer use for health	21 (28)		9 (26)		12 (29)		5 (19)		8 (32)		8 (35)	
	Computer use for diabetes	18 (24)		6 (18)		12 (29)		3 (11)		7 (28)		8 (35)	
	Wearable use for health	8 (11)		7 (21)		1 (2)		4 (15)		3 (12)		1 (4)	
	Wearable use for diabetes	7 (9)		6 (18)		1 (2)		4 (15)		2 (8)		1 (4)	
**Media use for health and diabetes management**													
	Texting use for health	19 (25)		13 (38)		6 (15)		8 (30)		6 (24)		5 (22)	
	Texting use for diabetes	18 (24)		11 (32)		7 (17)		6 (22)		6 (24)		6 (26)	
	Mobile app use for health	20 (27)		14 (41)		6 (15)		9 (33)		7 (28)		4 (17)	
	Mobile app use for diabetes	15 (20)		12 (35)		3 (7)		8 (30)		5 (20)		2 (9)	
	Email use for health	25 (33)		14 (41)		11 (27)		8 (30)		11 (44)		6 (26)	
	Email use for diabetes	22 (29)		12 (35)		10 (24)		6 (22)		10 (40)		6 (26)	
	Social media use for health	7 (9)		5 (15)		2 (5)		6 (22)		1 (4)		0 (0)	
	Social media use for diabetes	4 (5)		3 (9)		1 (2)		3 (11)		1 (4)		0 (0)	
	Podcast use for health	4 (5)		2 (6)		2 (5)		2 (7)		4 (16)		0 (0)	
	Podcast use for diabetes	1 (1)		1 (3)		0 (0)		1 (4)		0 (0)		0 (0)	
	Web video use for health	19 (25)		6 (18)		13 (32)		3 (11)		8 (32)		8 (35)	
	Web video use for diabetes	15 (20)		4 (12)		11 (27)		1 (4)		8 (32)		6 (26)	
	Website use for health	28 (37)		10 (29)		18 (44)		7 (26)		9 (36)		12 (52)	
	Website use for diabetes	28 (37)		12 (35)		16 (39)		9 (33)		9 (36)		10 (43)	

**Table 5:** Perceived value of a digital app to help with diabetes management (n=75).

			Total, n (%)		Type 1 diabetes (n=34), n (%)		Type 2 diabetes (n=41), n (%)		Aged <45 years (n=27), n (%)		Aged 45-59 years (n=25), n (%)		Aged >59 years (n=23), n (%)
Mobile app would be interesting			58 (77)		31 (91)		27 (66)		26 (96)		18 (72)		14 (61)
Mobile app would be helpful			58 (77)		30 (88)		28 (68)		25 (93)		18 (72)		15 (65)
Mobile app would be time saving			50 (67)		25 (74)		25 (61)		21 (78)		16 (64)		13 (57)
Confident that they could use an app			60 (80)		32 (94)		28 (68)		25 (93)		21 (84)		14 (61)
Likely to use an app			58 (77)		33 (97)		25 (61)		26 (96)		20 (80)		12 (52)
**App features participants were interested in**													
	Tracking blood sugar levels	64 (85)		30 (88)		34 (83)		25 (93)		21 (84)		18 (78)	
	Communicating with health professionals	60 (80)		29 (85)		31 (76)		24 (89)		21 (84)		15 (65)	
	Diet planning or tracking	57 (76)		23 (68)		34 (83)		21 (78)		18 (72)		17 (74)	
	Physical activity planning or tracking	57 (76)		30 (88)		27 (66)		25 (93)		17 (68)		15 (65)	
	SMS text message monitoring, notifications, and reminders	57 (76)		27 (79)		30 (73)		22 (81)		18 (72)		17 (74)	
	Communicating with others who have diabetes	45 (60)		21 (62)		24 (59)		18 (67)		16 (64)		11 (48)	

#### Qualitative Findings Related to Technology Use

Individuals who participated in the IDIs included 83% (10/12) female and 17% (2/12) male patient participants and 6 HCPs. Patient participant ages ranged from 21 to 66 years; 58% (7/12) were aged <40 years. Of the 12 participants, 11 (92%) had been diagnosed with T1D, and 1 (8%) female patient participant had a T2D diagnosis; all used insulin. While 17% (2/12) of the patient participants had private insurance, 25% (3/12) were uninsured at the time of the study, and the rest (7/12, 58%) had federal insurance coverage. The HCPs represented a range of specialists who are instrumental in the care of people with diabetes, including an endocrinologist, certified diabetes educator, certified medical assistant, and pharmacist (additional demographics are not provided to avoid identifying participants).

During the IDIs, patients and HCPs were asked about the use of apps for diabetes self-management. A total of 67% (4/6) of the HCPs and half (6/12, 50%) of the patient participants discussed the use of apps, with CalorieKing, MyFitnessPal, and Noom being mentioned by name. In total, 50% (3/6) of the HCPs indicated that they recommend use of CalorieKing to their patients, which can help with nutrient and meal tracking, including carbohydrate counting. One patient participant who was using CalorieKing at the time of the interview found it useful to be able to access existing food nutrition references for each serving and restaurant menu item rather than inputting all the information for her meals by hand. Another app, MyFitnessPal, was mentioned by 33% (2/6) of the HCPs as being sometimes recommended by themselves or their colleagues. One patient interviewee particularly enjoyed its barcode scanner feature to log nutrition information automatically. Some patient participants reported having used other apps specifically for diabetes self-management (eg, MyDiabetes); however, while they remembered features that allowed them to track blood glucose levels, meals, and physical activity, they could not remember the names of the apps, nor were they still using them. In addition, those who used CGM systems referred to using the associated CGM apps to review and share data with HCPs.

### CGM Use

#### Survey Findings

A total of 56% (42/75) of the participants reported having used a CGM device, but only 29% (22/75) were using one at the time of data collection (Table 6). The most commonly tested CGM model was Abbott’s FreeStyle Libre (no distinction was made during data collection between the different FreeStyle Libre models, namely, the 14-day system and FreeStyle Libre 2, the models available at the time of data collection), which was used by 81% (34/42) of all individuals with a history of CGM use and was most common across diabetes types and age groups. However, our initial use of phone-based recruitment of individuals with a LibreView profile may have led to a minor oversampling of participants with experience using the FreeStyle Libre. Dexcom’s G5 or G6 models were used by 8% (6/75) of the participants, all with T1D. On the basis of the estimated age of the smartphones that participants reported having and release dates of compatible operating systems, it seems unlikely that compatibility between CGM systems and phones would have been a barrier to use for many. CGM use history and current use differed by diabetes type and age—CGM use was more common among individuals with T1D (29/34, 85% had a history of use, and 18/34, 53% reported current use) than among those with T2D (13/41, 32% had ever used CGM, and 4/41, 10% reported current use). For those reporting current CGM use (22/75, 29%), the most commonly selected reason for using a CGM was “to instantly check my blood sugar” (19/22, 86%), followed by “helps me to have better control over blood glucose levels (by looking at trend arrows)” (17/22, 77%) and “to prevent low/high blood sugar faster” and “to see my sugar levels at times when checking may be harder (e.g. while sleeping)” (15/22, 68% in both cases).

The most common reasons for discontinuation of use centered on cost and issues with the sensor adhesive. Of the 20 individuals who reported reasons for discontinuing CGM use, 7 (35%) indicated that the device was too expensive, and 2 (10%) indicated that their insurance would not cover CGM (the latter was reported through an open-ended “specify other reasons” answer option). For 30% (6/20) of participants who had discontinued using a CGM, the device did not stick to the skin or caused a rash. Technical issues such as the device losing signal or malfunctioning accounted for the remaining barriers to continued use.

Among individuals who had no experience with CGM (33/75, 44%), the most common reason given was that they had never been offered the option (21/33, 64%). Other reasons given were the prohibitive cost of the device (4/33, 12%), not finding a comfortable and discrete place on the body for the sensor (1/33, 3%), a concern about infection for one individual who reported having a high risk of infection, finding the device too complicated to use (1/33, 3%), not being “technology savvy” (1/33, 3%), and “monitoring myself” (1/33, 3%).

**Table 6:** Self-reported continuous glucose monitoring (CGM) use and reasons for use and nonuse by diabetes type and age (n=75).

			Total, n/N (%)		Type 1 diabetes, n/N (%)		Type 2 diabetes, n/N (%)		Aged <45 years, n/N (%)		Aged 45-59 years, n/N (%)		Aged >59 years, n/N (%)
History of CGM device use			42/75 (56)		29/34 (85)		13/41 (32)		22/27 (81)		13/25 (52)		7/23 (30)
Used Abbott FreeStyle Libre model			34/42 (81)		23/29 (79)		11/13 (85)		19/22 (86)		10/13 (77)		5/7 (71)
Used Dexcom G5 or G6			6/42 (14)		6/29 (21)		0/13 (0)		1/22 (5)		3/13 (23)		2/7 (29)
Current CGM use			22/75 (29)		18/34 (53)		4/41 (10)		13/27 (48)		5/25 (20)		4/23 (17)
**Reasons for current CGM use**													
	Respondents, n	22/75 (29)		18/34 (53)		4/41 (10)		13/27 (48)		5/25 (20)		4/23 (17)	
	To instantly check my blood sugar	19/22 (86)		15/18 (83)		4/4 (100)		12/13 (92)		4/5 (80)		3/4 (75)	
	Helps me have better control over my blood glucose levels (by looking at trend arrows)	17/22 (77)		15/18 (83)		2/4 (50)		10/13 (77)		4/5 (80)		3/4 (75)	
	To prevent low/high blood sugar faster	15/22 (68)		14/18 (78)		1/4 (25)		8/13 (62)		4/5 (80)		3/4 (75)	
	To see my sugar levels at times when checking may be harder/ (eg, while sleeping)	15/22 (68)		14/18 (78)		1/4 (25)		9/13 (69)		4/5 (80)		2/4 (50)	
	My health care provider recommended I use it	14/22 (64)		13/18 (72)		1/4 (25)		9/13 (69)		3/5 (60)		2/4 (50)	
	Helps me lower my glycated hemoglobin levels	13/22 (59)		12/18 (67)		1/4 (25)		7/13 (54)		4/5 (80)		2/4 (50)	
	Makes me feel safer	13/22 (59)		12/18 (67)		1/4 (25)		9/13 (69)		2/5 (40)		2/4 (50)	
	Gives me/my health care provider useful information that can help make insulin requirements more accurate	12/22 (55)		12/18 (67)		0/4 (0)		7/13 (54)		3/5 (60)		2/4 (50)	
	To have a better understanding of how insulin, food, or physical activity change my blood sugar	11/22 (50)		10/18 (56)		1/4 (25)		6/13 (46)		2/5 (40)		3/4 (75)	
	Gives me more motivation to make healthier choices	11/22 (50)		11/18 (61)		0/4 (0)		6/13 (46)		3/5 (60)		2/4 (50)	
**Reasons for discontinuation of use**													
	Respondents, n	20/75 (27)		11/34 (32)		9/41 (22)		9/27 (33)		8/25 (32)		3/23 (13)	
	Device is too expensive	7/20 (35)		3/11 (27)		4/9 (44)		2/9 (22)		4/8 (50)		1/3 (33)	
	Device/adhesive gives me a rash or other skin problem	1/20 (5)		1/11 (9)		0/9 (0)		1/9 (11)		0/8 (0)		0/3 (0)	
	Device/adhesive does not stick to my skin	5/20 (25)		4/11 (36)		1/9 (11)		3/9 (33)		2/8 (25)		0/3 (0)	
	Device loses signal/does not function well	4/20 (20)		2/11 (18)		2/9 (22)		1/9 (11)		2/8 (25)		1/3 (33)	
	Insurance issues	2/20 (10)		0/11 (0)		2/9 (22)		0/9 (0)		1/8 (13)		1/3 (33)	
**Reasons for no previous CGM use**													
	Respondents, n	33/75 (44)		5/34 (15)		28/41 (68)		5/27 (19)		12/25 (48)		16/23 (70)	
	Never been offered to me	21/33 (64)		1/5 (20)		20/28 (71)		1/5 (20)		9/12 (75)		11/16 (69)	
	Device is too expensive	4/33 (12)		3/5 (60)		1/28 (4)		3/5 (60)		1/12 (8)		0/16 (0)	
	Hard to find a comfortable and discrete place on my body to place the device	1/33 (3)		1/5 (20)		0/28 (0)		1/5 (20)		0/12 (0)		0/16 (0)	
	Device is too complicated to use	1/33 (3)		0/5 (0)		1/28 (4)		0/5 (0)		1/12 (8)		0/16 (0)	
	Concerned about contracting an infection	1/33 (3)		0/5 (0)		1/28 (4)		0/5 (0)		0/12 (0)		1/16 (6)	
	Was just offered one	1/33 (3)		1/5 (20)		0/28 (0)		1/5 (20)		0/12 (0)		0/16 (0)	
	Not technology savvy	1/33 (3)		0/5 (0)		1/28 (4)		0/5 (0)		0/12 (0)		1/16 (6)	
	I monitor myself	1/33 (3)		0/5 (0)		1/28 (4)		0/5 (0)		0/12 (0)		1/16 (6)	

#### Qualitative Findings Related to CGM Use

During interviews, the overwhelming sense across both patients and HCPs was that CGM devices were highly beneficial additions to the suite of diabetes self-management tools. All patient participants referenced how easily they could check their blood glucose repeatedly during the day, even discretely in public. They mentioned the convenience of the device allowing them to check their blood glucose at strategic time points (eg, on waking, before and after meals, and when periodically alerted to potential hypo- and hyperglycemic events by alarm notifications), which made it possible for them to take preventive measures to avoid their blood glucose rising or falling too much. Patients also took advantage of the added convenience by simply checking their glucose more frequently throughout the day as they did not need to carry additional supplies or stick their finger to obtain a reading.

Despite checking their readings more often with their CGM system than when using finger sticks, only 33% (4/12) of the patient participants, all female, aged <40 years, and covered by federal insurance, mentioned leveraging the data to track patterns in their blood sugar levels. Each described different reasons for doing so. For example, one female participant in her late 30s with T1D generally liked having “more insight into what’s going on,” even at night. Another female participant of the same age with T1D talked about reviewing her data to understand her responses to certain foods and plan for her meals as she could anticipate how her body would react. A younger woman with T1D indicated that the data (supported by the alarms) had helped her significantly in taking preventive measures when her blood sugar level was decreasing. She indicated that she had learned about target time in range and used the summary data to make sure that she was keeping within her target percentage time in range.

Patient participants also highlighted the system’s ability to support their communication with HCPs by making their longitudinal data available to their care teams. This helped streamline some of the conversations during appointments as HCPs could review data ahead of time, and patient participants also appreciated the ability to obtain insights from HCPs when they saw unusual glucose activity and needed clarification between appointments.

The challenges to CGM use discussed by patient participants mirrored those reported in the survey—58% (7/12) of the participants mentioned the lack of insurance coverage rendering CGM use prohibitively expensive, and 33% (4/12) indicated that the adhesive failing to keep the sensor on the skin or the sensor falling off when bumped made its use impractical and the financial burden even larger. Others mentioned that the alarms waking them up at night or being loud and not shutting off quickly could be annoying, although one did note that the new model of the sensor she had previously used had optional alarms, which addressed her one complaint with the device.

HCPs echoed these sentiments as well, noting the barrier of cost and lack of insurance coverage. This was due to some patients’ individual treatment plans (eg, not using insulin) rendering them ineligible for continued insurance coverage for CGM systems and related supplies. However, they also mentioned that some patients, if they were financially able to, chose to pay for CGM supplies out of pocket due to the value they placed on the devices. HCPs were not asked to elaborate on the reasons why patients might not be offered CGM.

Among HCPs, the same benefits of reducing the burden of finger pricks and streamlining the process of checking blood glucose levels were echoed. One noted that the convenience was especially useful for individuals who need more frequent monitoring, such as those at higher risk of hypoglycemia, as the CGM allowed them to feel more confident taking their insulin while avoiding their blood glucose dropping too low. Other groups considered to particularly benefit from CGM included individuals with challenging-to-manage diabetes (often due to high glycemic variability); those with hypoglycemia unawareness; and, more generally, individuals with a T1D diagnosis.

Another benefit discussed by HCPs, which aligned with patient participants’ comments, was the value of being able to add explanatory notes to specific times of day or events to help HCPs and patients themselves better understand the reports generated by the CGM systems. The data helped fill in gaps for HCPs and support clinical decision-making for patients who might not normally check their blood sugar frequently. Although this value of the data was universally discussed by HCPs, they did agree that patients generally checked the main glucose reading at individual time points rather than engaging with the patterns that emerged over longer use. One HCP reported that some of her patients did “their own little experiments” when they changed their eating or exercise habits and tracked the effects via the CGM data.

In addition, according to HCPs, patients tended to prefer the convenience of using a phone-based CGM app rather than a separate reader; however, they pointed out that, for patients without a compatible phone or without a reliable internet connection, the ability to share data with an HCP or to track trends was reduced as they could not easily upload the data before appointments, nor could they leverage the longitudinal data themselves between health care visits.

## Discussion

### Principal Findings

This study found that patients receiving care for diabetes at an inner-city safety-net hospital had access to a range of technology communication devices, with almost universal smartphone and home Wi-Fi access. While some also had access to other types of communication devices and used a range of media platforms and two-thirds of the participants (51/75, 68%) reported use of their smartphones for general health or diabetes management, the use of specific digital tools for those purposes remained low. CGM systems were seen by patient participants and HCPs as positive enhancements to diabetes self-management, helping patients take preventive measures and better achieve glycemic control. There remain opportunities for patients to better leverage their access to both digital communication technologies and, when available, CGM to enhance their diabetes self-management.

### Comparisons With Prior Work

While innovative technologies continue to transform diabetes care and self-management, equity concerns persist. Pilot studies have demonstrated the potential of mobile apps to improve diabetes outcomes [26]; however, concerns have been raised about the risk of increasing digital marginalization and health disparities if efforts are not made to ensure equitable access for socially disadvantaged groups [27].

Our findings align with those from other studies that have reported a reduction in the digital divide in terms of access to devices connected to the internet (particularly smartphones) [16]. We found that the vast majority of patients receiving care at an inner-city safety-net hospital in Atlanta, Georgia (71/75, 95%), owned a smartphone compared to 85% of the US population (where rates are lowest among those aged >65 years, 61%; those with the lowest socioeconomic status, 76%; and educational levels, 75%; and those living in rural areas, 80%) [15].

However, the use of available technologies for health management appears to be limited among underresourced patients. For example, the use of apps for health purposes among participants in our study was low (20/75, 27%). According to a 2015 Pew Research Center study, over half of mobile phone users have downloaded a mobile health (mHealth)–related app, with fitness and nutrition apps being the most popular [28].

Interest among participants in our study in an app to help them manage their diabetes was high, along with related self-efficacy and perceived likelihood of using such an app. Three-quarters of the participants had an unlimited smartphone data plan (55/75, 73%), and 89% (67/75) had internet access at home, suggesting that logistical barriers to app use would be limited.

Our findings echo those from a community-based survey of a vulnerable, low-income, predominantly Latino population (n=104) in East Harlem, New York [16], which found the digital divide to be narrowing. While a higher proportion of smartphone owners (39%) in the New York study than in our study reported using health apps, those who had graduated high school were 7 times more likely to use health apps than those who had not, suggesting a potential link to income or health literacy. Our study did also observe higher app use and interest in a diabetes self-management support app among high school graduates. As in our study, participants in the study by Vangeepuram et al [16] expressed interest in a health promotion app. Our data also align with findings from an earlier study focused on the appeal of an mHealth diabetes app and conducted in both a medically underserved area and a more affluent suburb [29]. That 2015 study also found that younger individuals were more likely to be interested in mHealth solutions than their older counterparts while also providing support for people from underserved communities being particularly interested in using mHealth apps.

Participants in our study who had experience with CGM reported high levels of satisfaction with the experience. However, most participants in our study (53/75, 71%) did not have the opportunity to benefit from CGM, either because they had not been offered CGM by HCPs (as noted, we did not explore reasons for this with HCPs during IDIs) or had had to discontinue use due to a lack of insurance coverage and the prohibitive cost of the sensors. These factors, in conjunction with previously reported data indicating lower rates of CGM use and high rates of discontinuation among African American individuals, our primary population in this study, point to a need to increase the accessibility and affordability of these tools [5-9,30]. In parallel, it is crucial to address well-documented implicit HCP biases resulting in lower rates of CGM recommendation and prescription to patients from racial or ethnic minority groups and other populations vulnerable to health disparities [6,31-33]. There have been efforts to expand the use of CGM, and the evidence for its use among different demographics, including those with T2D, is growing [34-39]. Health systems, payers, and policy makers should continue efforts to increase technology access for underresourced communities to promote more equitable access to the diabetes digital ecosystem [40].

In addition to patients having limited access to CGM systems, there is an underuse of CGM data and visualizations by those who do use CGM. HCPs in this study discussed their patients making little use of CGM data, and only a minority of the patient participants indicated that they engaged with the data patterns. While it is unclear whether this was due to a lack of interest in the patterns or a gap in knowledge about the data, other studies have found that, without additional support, CGM data remain challenging to interpret by users [34], reducing the potential of these data to effect behavior change and lead to glycemic control among users, even with increased access to CGM systems. This and existing studies of CGM training programs demonstrate the important role of increasing educational resources related to the use of CGM data to enhance and support glycemic control [41-46].

Our participants’ expressed willingness, confidence, and self-efficacy for the use of a diabetes management app point to the likely acceptability and feasibility of such an app to increase diabetes self-management skills among low-income individuals with diabetes. However, to ensure uptake and maximize the effectiveness of such an app, it is imperative that it be designed in tandem with people living with diabetes, their HCPs, and other relevant stakeholders. This would also help avoid the current challenges of existing commercially available diabetes management apps, many of which are overly complex or unappealing [19-21]. While alternatives to an app, such as a website or informational web-based portal, exist, as current CGM systems report data through apps, we anticipate that this platform may be more familiar and integrate better into participants’ lives.

### Limitations

Limitations of the study include the relatively small convenience sample, the disproportionate number of female participants despite efforts to enroll male participants, and potential bias toward those more comfortable with technology. Individuals who felt less technologically literate may have been less interested in completing a survey about technology, especially as the survey itself was either offered via emailed link to those recruited by phone or presented on a tablet to those recruited on-site at the clinic. Offering a paper version of the survey may have increased the representation of individuals less at ease with technology. In addition, our initial phone-based recruitment of individuals with a LibreView profile may have led to a minor oversampling of participants with experience using the FreeStyle Libre relative to other CGM systems, although only 5% (4/75) of participants from our sample were recruited via this method. Finally, while the most common reason patients gave for never having used CGM was lack of HCP recommendation, their eligibility for CGM was not validated through medical record review, nor was this topic explored with HCPs.

### Conclusions

Although access to digital communication technologies was widespread among patients receiving care for diabetes at an Atlanta, Georgia, safety-net hospital’s diabetes center, those same patients were not taking full advantage of those technologies to support their diabetes care. Among participants with experience using CGM systems, satisfaction was high, although there was potential to further increase the benefits of this technology through additional support to boost CGM access and diabetes technology literacy. There was strong acceptability and likelihood of use of a digital app for diabetes management support. These findings, in combination with the anticipated increased access to smartphones and CGM systems, support further research into the development of innovative digital solutions to support diabetes management.

## Abbreviations

CGM: continuous glucose monitoring
GDC: Grady Diabetes Center
HCP: health care provider
IDI: in-depth interview
mHealth: mobile health
REDCap: Research Electronic Data Capture
T1D: type 1 diabetes
T2D: type 2 diabetes
